# Whose data is it anyway? Patient experience and service improvement

**DOI:** 10.1177/1355819620921423

**Published:** 2020-06-15

**Authors:** Glenn Robert, Sara Donetto

**Affiliations:** 1 Professor of Health care Quality & Innovation, Florence Nightingale Faculty of Nursing, Midwifery & Palliative Care, King’s College London, UK; 2 Lecturer, Florence Nightingale Faculty of Nursing, Midwifery & Palliative Care, King’s College London, UK

In this issue, Locock et al. report on an ethnographic study of how frontline staff in six NHS hospitals in England might use patient experience data to improve the quality of health care.^1^ The study findings are an important contribution to understanding the practical, everyday work undertaken by teams of frontline staff to improve the quality of the care and services they provide. They also suggest how staff might be better supported to work towards person-centred quality improvement. As one of the nine empirical studies exploring the use of patient experience data which were funded by the National Institute for Health Research Health Services & Delivery Research programme, this study also contributes to the wider consensus that there are gaps in health care providers’ capacity to analyse and use patient experience data.^2^

A stark finding from Locock et al. is that frontline staff were unsure as to what counted as patient experience data and how these could be found and used in quality improvement projects on the participating medical wards. This uncertainty is significant especially in view of policy initiatives to raise the profile and importance of using such data.^3^ Indeed, five of the six teams studied appear to have felt the need to generate new data to inform their projects, despite what has been termed ‘an explosion in the collection of feedback from patients about their opinion of health care services throughout many countries across the world’.^4^ This raises the question of why, with so much patient experience data being collected both nationally and locally, staff felt they had to generate additional sources of information? Staff in Locock et al.’s study saw surveys as the most immediately recognizable source of data but considered qualitative data (for example, patient stories, free-text comments in surveys and online patient feedback) more reflective of lived experiences and therefore appealing. However, they were unsure as to how to use these latter forms of data in their projects. This suggests a continuing mismatch between the features of existing data collection methods and resulting data and the aims and information needs of staff trying to undertake quality improvement work at ward level.

A further key insight from the study is the variation in the composition of the teams formed at each of the six participating hospitals to design, carry out, evaluate and report on ward-specific improvement projects. As the authors remark, the variation in composition suggested that ‘there wasn’t an accepted home for patient experience improvement within these organizations.’ Locock et al. usefully highlight how the ways the teams were convened came to comprise different combinations of the four forms of capital identified by sociologist and public intellectual Pierre Bourdieu^5^: economic (in this context, for example, office/ward equipment), social (clinical/non-clinical networks), symbolic (reputation and status of team members) and cultural (expertise in particular forms of practice). The authors refer to the various assortments of these forms of capital in the improvement projects as ‘team capital’. More diverse teams appeared to make more progress with their projects. But noticeably – and despite recent descriptions of a pervasive ‘new spirit of participation’ in health care^6^ – patient input appears to have been limited to the early stages of only two of the six projects examined. How might including patients throughout as project members have supplemented the types of ‘team capital’ identified? The study findings therefore raise questions relating to the decision-making processes which underpinned the formation of the teams and the assumptions which guided them.

Locock et al. also point to the ongoing conflation between data for performance management and data for quality improvement. Despite calls for culls of nationally imposed metrics and targets in the US,^7^ the formal collection of patient experience data continues to support a small industry of ‘data mining’, ‘machine learning’ and ‘maximizing response rates’ that is dominated in England by (still) mandatory indicators such as the Friends & Family Test.^8^ This is often at the expense of valuing other forms of data and, as the authors note, this narrow focus may result in ‘potentially useful intelligence [being] disregarded’. Such ‘soft intelligence’ – the processes and behaviours associated with seeking and interpreting data that evades easy capture and quantification – is not only directly relevant to care improvement but can also help question taken-for-granted assumptions about quality, safety and organizational performance.^9^ Consequently, Locock et al. call for a ‘shift from top-down measurement for performance to an approach which embraces frontline wisdom and creativity, and involves a broad coalition of staff in improvement work’.^1(p.9)^ Whilst Locock et al. acknowledge the challenge of finding ‘ways to encourage staff empathy and creativity as a route to patient experience without reverting to assumptions that staff know best’,^1(p.5)^ the importance of granting ownership, autonomy and resources to frontline staff for quality improvement is a consistent message from other empirical studies.^2,4,10^

Structures and systems for patient experience data collection and use should therefore be aligned with formal quality improvement, whilst also recognizing the equal value of the everyday, informal nature of much quality improvement work on wards,^10^ particularly in relation to patient experience.^2^ Health care organizations need to understand and make sense of the different combinations of ways in which they currently ask for feedback and what they do with that feedback. To help encourage more useful feedback in the future, organizations should establish local mechanisms that embed action as a result of feedback and demonstrate how that feedback improves both patient and staff experiences.

At a more fundamental level, we would argue that many, if not all, of the issues above point towards a need to create and support new spaces in which patients and staff can relate more closely to each other’s experiences. We have previously argued that formal approaches to improving patient experience ‘continue to be hindered by a deeply engrained perception of patients and families as passive sources of data rather than active partners in implementing change’.^11(p.2)^ Consistent with recent calls for a new era of health care quality improvement,^7^ the spaces we refer to would view patients (and staff) not solely as sources of data but as active participants working together in ongoing efforts to improve their experiences of a service.

This may require extending Locock et al.’s important findings about team capital to include patients and citizens. Such a reconfiguration of teams working on improving quality would certainly require some ‘infrastructuring’ in the sense of designing situations and materials which enable new forms of discussions and activities to take place,^12^ but – more crucially – a change in mindset. A new mindset would involve critiquing current approaches to the collection and use of patient experience data in terms of ‘potentially humanizing and dehumanizing elements’ and translating them into practice ‘in ways that place people as human beings at the centre of care’.^13(p.68)^ This will require a much more continuous and relational approach to quality improvement with forms of involvement of staff and patients that could build on the ‘rich history of research in the fields of community-engaged research, participatory and collaborative practice’.^14(p.12)^ It will also require organizations to acknowledge and value staff’s practical wisdom whilst developing and consolidating enabling processes and systems that allow patients’ voices to be heard directly.
